# Supplementary material for: Nebulized Lipopolysaccharide Causes Delayed Cortical Neuroinflammation in a Murine Model of Acute Lung Injury
Source: Int J Mol Sci. 2024 Sep 20;25(18):10117. doi: 10.3390/ijms251810117 (PMC11432715; doi:10.3390/ijms251810117)
Supplement: Supplementary file 1 [file ijms-25-10117-s001.zip › ijms-3201876-supplementary.pdf]

Supplementary Materials

Table S1. Detailed results of mRNA expression normalized to PPIA (brain). * p < 0.05, and *** p < 0.001

	Cortex	*p* value	Hippocampus	*p* value
		IL-6		
	**Cortex**	***p* value**	**Hippocampus**	*p* value
veh	1.857e-005±1.338e-006		1.093e-005±4.275e-007	
24h	1.778e-005±1.646e-006		9.941e-006±5.936e-007	
48h	2.001e-005±1.269e-006		9.591e-006±4.843e-007	
72h	2.035e-005±1.26oe-006		1.033e-005±4.197e-007	
96h	1.754e-005±1.129e-006		9.199e-006±3.861e-007	
		GFAP		
	Cortex	*p* value	Hippocampus	*p* value
veh	0.05294±0.003562	*0.0001* (72h) ***	0.003016±0.0004107	
		0.0018 (96h) ***		
24h	0.03232±0.003791	*<0.0001* (72h) ***	0.002221±0.0002014	
		<0.0001 (96h) ***		
48h	0.069078±0.01245	*0.0001* (72h) ***	0.002446±0.0001262	
	0.1220±0.01209	*0.0018* (96h) ***		
72h	0.1119±0.008030		0.002979±0.0003447	
96h			0.002538±0.0001922	
		c-Fos		
	Cortex	*p* value	Hippocampus	*p* value
veh	0.01050±0.001472	*0.0031* (72h) ***	0.01358±0.003149	
		0.0013 (96h) ***		
24h	0.00929±0.001118	*0.0003* (72h) ***	0.01281±0.001846	
		0.0001 (96h) ***		
48h	0.01057±0.002085	*0.0009* (72h) ***	0.01108±0.001016	
		0.0003 (96h) ***		
72h	0.02434±0.003548		0.01529±0.001973	
96h	0.02598±0.002422		0.01140±0.001787	
		Gadd45b		
	Cortex	*p* value	Hippocampus	*p* value
veh	0.007949±0.0004740	*0.0180* (96h) *	0.01151±0.000922	
24h	0.006585±0.0004666	*0.0007* (72h) ***	0.0150±0.0004470	
		<0.0001 (96h) ***		
48h	0.007134±0.0006283	*0.0088* (72h) ***	0.009462±0.0003152	
		0.0002 (96h) ***		*0.0022* (72h) ***
72h	0.009447±0.0004457		0.01197±0.0005557	*0.0314* (96h) *
96h	0.01031±0.0004296		0.01141±0.0004849	
		Arc		
	Cortex	*p* value	Hippocampus	*p* value
veh	0.006956±0.001102	*0.0455* (72h) *	0.005947±0.0004377	
		0.0455 (96h) *		
24h	0.005899±0.0008328	*0.0030* (72h) *	0.007498±0.0007832	*0.0040* (72h) ***
		0.0030 (96h) *		*0.0681* (96h)
48h	0.005617±0.001180	*0.0028* (72h) *	0.006752±0.0004710	*0.0225* (72h) *
		0.0028 (96h) *		
72h	0.01261±0.001738		0.01147±0.001300	*0.0052* (72h) ***
96h	0.01268±0.001403		0.009871±0.001061	
		TLR4		
	Cortex	*p* value	Hippocampus	*p* value
veh	0.00037±2.684e-005	*0.0480* (72h) *	0.00037±2.684e-005	
24h	0.00036±2.051e-005	*0.0048* (72h) ***	0.00036±2.051e-005	
48h	0.00041±2.200e-005		0.00041±2.200e-005	
72h	0.00047±2.612e-005		0.00047±2.612e-005	
96h	0.00044±2.021e-005		0.00043±2.021e-005	
